# Potential Clinical Benefits of D-ribose in Ischemic Cardiovascular Disease

**DOI:** 10.7759/cureus.2291

**Published:** 2018-03-09

**Authors:** Linda M Shecterle, Kathleen R Terry, John A St. Cyr

**Affiliations:** 1 Research and Development, Jacqmar, Inc.

**Keywords:** d-ribose, adenine nucleotides, cardiovascular disease, cardiology, cardiovascular surgery, clinical benefits, adenosine triphosphate, pre-clinical and clinical trials

## Abstract

Cardiovascular disease still remains the leading cause of deaths worldwide. Atherosclerosis, the most common type of cardiovascular disease, has continued to progress due to many factors, genetics, and lifestyles. All cells require adequate adenosine triphosphate (ATP) levels to maintain their integrity and function. Myocardial ischemia commonly found in atherosclerosis can produce lower levels of ATP, which affects not only cellular energy, but also alters normal function. D-ribose, a naturally occurring pentose carbohydrate, has been shown to increase cellular energy levels and improve function following ischemia in pre-clinical studies and have demonstrated potential benefits in clinical evaluations. This review paper presents an overview of ischemic cardiovascular disease and the potential role that D-ribose could play in improving myocardial energy levels and function in the area of ischemic cardiovascular diseases.

## Introduction and background

Cardiovascular disease still remains the leading cause of deaths worldwide for both men and women. There are approximately 61 million individuals afflicted with heart disease in the United States according to the Centers for Disease Control and Prevention. The World Health Organization has reported that approximately 29% of all deaths worldwide are related to this disease state [1]. Atherosclerosis or arterial intimal plaque formation has been shown to progress over time due to factors such as genetics and lifestyles. Obviously, trying to modify genetic factors is not practical; however, changes in lifestyle are plausible. Major lifestyle factors can include consuming a non-healthy diet, smoking, hypertension, lack or inadequate amount of exercise, and potentially continuous stress. Constant efforts have continued to adequately address and to stress to patients that making lifestyle changes could play a positive role in the lower progression of this disease and thereby potentially lower their risk of a cardiovascular event. Precisely, the medical profession has instructed their patients to alter their lifestyle by changing to a healthier diet, regular exercise, cessation of smoking, control of blood pressure, and seek efforts to lower stress.

Increasing plaque formation can ultimately limit the delivery of blood and thereby oxygen to viable tissues. Atherosclerosis is not confined to a sole anatomic region, for it is a systemic disease that can involve various arterial regions, such as the heart, cerebrovascular, and peripheral circulation. Clinically significant coronary arterial atherosclerotic disease can produce symptoms of angina or chest pain, commonly during and following stressful situations; however, some patients can even have symptoms at rest. Furthermore, with the progression of coronary artery disease with increasing atherosclerosis plaque formation, these patients are susceptible to sustaining an acute myocardial infarction or live with a chronic, debilitating condition, which over a prolonged period of time, can potentially advance into the development of congestive heart failure. Coupled with coronary artery ischemia and with longstanding hypertension, many patients develop myocardial diastolic dysfunction, an abnormality in ventricular relaxation. Ventricular dysfunction has continued to be a challenging clinical problem because, as of today, there is not yet an approved, solely directed therapy, both pharmaceutical and device-related, targeted at effectively treating this myocardial dysfunctional state [1].

Medical investigations have found that left ventricular diastolic dysfunction is more prevalent than expected. Redfield et al. stated that “systolic dysfunction is frequently present in individuals without cognized congestive heart failure. Furthermore, diastolic dysfunction is common, often not accompanied by recognized congestive heart failure, and is associated with marked increases in all-cause mortality.” In 2003, Redfield et al. reported in randomly selected adults over the age of 45 that 21% had mild and 7% had at least moderate diastolic dysfunction. Six percent had moderate or severe diastolic dysfunction with a normal ejection fraction [2]. Bursi et al. found that “more than half of patients with heart failure have preserved ejection fraction, and isolated diastolic dysfunction is present in more than 40% of cases" [3]. Fischer et al. found diastolic dysfunction in 2.8% of individuals between 25-35 years of age with an increased incidence (15.8%) in individuals older than 65 years of age [4]. Compared to women, men had a higher rate of diastolic abnormalities (13.8% vs. 8.6%). Associated factors responsible for the development of diastolic abnormalities have included longstanding hypertension, evidence of left ventricular hypertrophy, and coronary artery disease. Additionally, diastolic dysfunction has been related to a high body mass index, high body fat, and individuals with diabetes mellitus.

Myocardial ischemia alters oxidative cellular metabolism, reflected in lower levels of adenosine triphosphate (ATP). Different states of ischemia, transient versus chronic, can reflect varying degrees of cellular reductions in ATP levels, as well as functional abnormalities and altered cellular homeostasis, and if severe enough, cellular viability. Myocardial ischemia is a common underlying cause in patients with congestive heart failure, and many of these patients also experience a degree of diastolic dysfunction. Clinically, at least 50% of patients diagnosed with congestive heart failure will have various degrees of diastolic dysfunction solely or potentially combined with systolic dysfunction. Within the last decade, Ingwall and Weiss proposed that the failing heart is energy starved [5]. This theory had been previously proposed but was not actively pursued because it was unclear whether ATP levels, in fact, were really decreased; if these levels did decrease in the failing heart, many physicians and scientists thought that the remaining pool of high energy phosphates should be sufficient enough to satisfy myocardial energy-requiring reactions. The advancement in biophysical tools, such as nuclear magnetic resonance spectroscopy and positron emission tomography scans, have aided in providing a better understanding of this myocardial energy/functional relationship.

## Review

Cellular bioenergetics and function

Every cell requires adequate levels of high-energy phosphates to maintain their integrity and function. This cellular supply of energy (precisely, ATP) usually meets demands. Adenosine triphosphate is produced by intracellular pathways, such as glycolysis and the tricarboxylic acid cycle, with glucose as the starting substrate. However, some cells can also rely on alternative pathways, such as the pentose phosphate pathway, for the production of ATP [6]. There is a direct relationship between adequate myocardial ATP levels and the development of ventricular diastolic dysfunction. Calcium plays a major role in this interaction. Adenosine triphosphate provides the energy for this interaction between cytosolic calcium and the sarcoplasmic reticulum. Depleted ATP levels can result in calcium remaining fixed to troponin longer in diastole, producing a state of diastolic dysfunction or altered ventricular compliance. Following ischemia, a return in diastolic function may be limited by the availability of the high-energy phosphate, ATP [7]. Theoretically, efforts to maximize the recovery of myocardial ATP levels during and following ischemia could aid in minimizing the functional untoward effects of ischemia.

When cells are subjected to ischemia or hypoxia, the normal production of energy compounds is reduced. Myocardial ischemia depletes ATP levels, which can affect intracellular reactions and the cells' function. There are degrees of ischemia, and if severe enough, the viability of the cell is jeopardized. Researchers have also demonstrated that this depletion in myocardial ATP levels following ischemia can last for a considerable time period due to a slow adenine nucleotide synthesis, which is reflected in an alteration in function [8]. Zimmer reported that an extended time period was required for the recovery of depressed myocardial energy levels, as well as an improvement in the alteration in mechanical function, following ischemia [9]. Many of these myocardial ischemic studies involved "isolated hearts", which have limitations due to the nature of the isolated, excised heart preparations. Therefore, an intact acute animal model has also been developed to assess and confirm the findings observed in the “isolated heart” models. Short-term investigations involving an intact animal model found similar findings in myocardial adenine nucleotide levels following ischemia. Reibel and Rovetto reported in isolated perfused rat hearts that a moderate ischemic insult resulted in a 50 - 70% decrease in myocardial ATP levels [10]. Similarly, Reimer et al. reported that 12 to 30 minutes of myocardial ischemia produced a substantial drop in ATP levels with a significant decrease in total adenine nucleotides, and days were required for total recovery [11]. However, to better appreciate this important energy-functional relationship following ischemia, a chronic animal model was developed to provide the means for long-term assessment of myocardial energy levels and function during and following ischemia [12-13], (Ward HB, Kriett J St.Cyr J, et al.: Relationship between recovery of myocardial ATP levels and cardiac function following ischemia. J Am Coll Cardiol. 1984, 3:544 abstr.). In this chronic canine model, 20 minutes of normothermic, global myocardial ischemia produced a significant 50% decrease in ATP levels, which was accompanied by a state of left ventricular diastolic dysfunction. Furthermore, the data from this chronic animal study confirmed that myocardial ischemia has a long-term effect. These researchers reported that there was a substantial delay, over one week, in myocardial energy levels and functional recovery following this moderate, reversible ischemic insult [12-13], (Ward HB, Kriett J, St.Cyr J, et al.: Relationship between recovery of myocardial ATP levels and cardiac function following ischemia. J Am Coll Cardiol. 1984, 3:544 abstr.).

Adenine nucleotide metabolism and D-ribose

**Figure 1:**
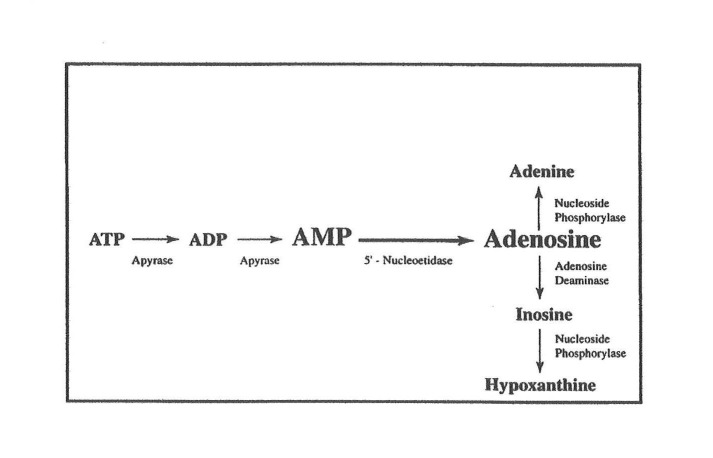
Catabolism of adenine nucleotides ATP: adenosine triphosphate; ADP: adenosine diphosphate; AMP:  adenosine monophosphate

Ward et al. reported that myocardial precursor availability is an important limiting factor in the recovery of myocardial ATP molecules following ischemia [13]. Adenine nucleotide catabolism eventually produces precursors, which can be lost from the cell, and therefore, inhibits recovery of ATP (Figure 1).

The ultimate repletion of myocyte ATP levels following ischemia depends on the availability of the precursor phosphoribosyl-pyrophosphate (PRPP), and D-ribose has shown to accelerate this nucleotide synthesis by directly increasing PRPP levels [14]. Various adenine nucleotides have been investigated for their potential to replenish post-ischemic lower levels of myocardial ATP, such as adenosine, 5-amino-4-imidazole carboxamide riboside, inosine, adenine, and D-ribose. Not only precursors have been evaluated, but also the use of adenine degradative enzymatic inhibitors for the maintenance or accelerated replenishment in energy levels following ischemia. The majority of these substrates and inhibitors have, at best, shown minimal improvement in both ATP recovery and function. However, D-ribose supplementation has been shown to offer great benefits. D-ribose, a naturally occurring pentose carbohydrate, has repeatedly demonstrated in numerous animal studies its significant benefit in enhancing the return in ATP levels and improving diastolic function following myocardial ischemia.

Metabolism of D-ribose

**Figure 2:**
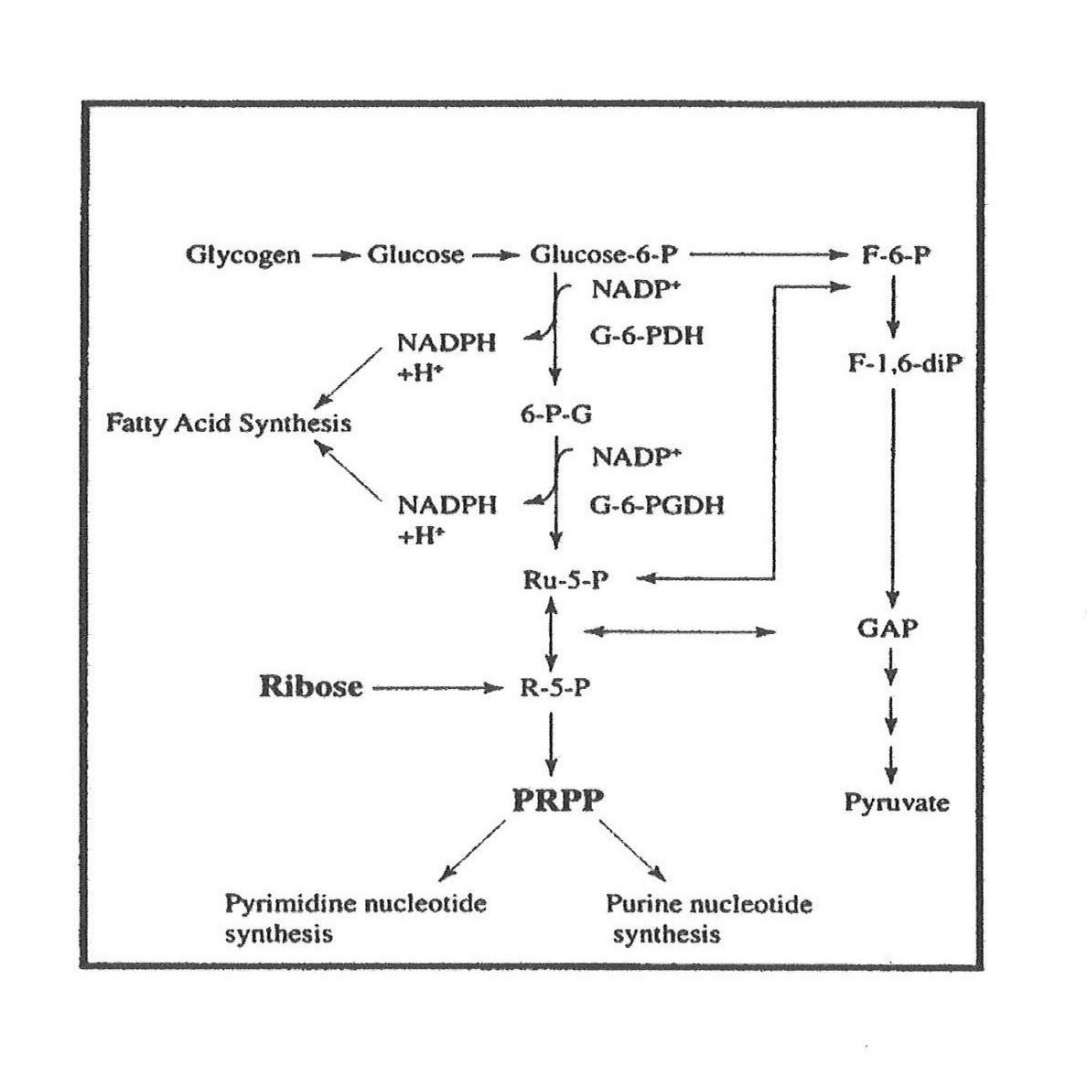
Pentose phosphate pathway F-6-P: fructose-6-phosphate; F-1,6-diP: fructose-1,6-diphosphate; GAP: GTPase activating protein; G-6-PDH: glucose-6-phosphate dehydrogenase; G-6-PGDH: glucose-6-phosphogluconate dehydrogenase; NADP+: nicotinamide adenine dinucleotide phosphate; NADPH + H: Nicotinamide adenine dinucleotide phosphate (reduced form) plus hydrogen; NADPH: nicotinamide adenine dinucleotide phosphate; PRPP: phosphoribosyl-pyrophosphate; R-5-P: ribose-5-phosphate; Ru-5-P: ribulose-5-phosphate; 6-P-G: 6-phosphogluconate

D-ribose can produce nucleotides and metabolic intermediates, such as ribose-5-phosphate. Ribose-5-phosphate is an important intermediate of the pentose phosphate pathway, also known as the hexose monophosphate shunt or phosphogluconate pathway (Figure 2).

**Figure 3:**
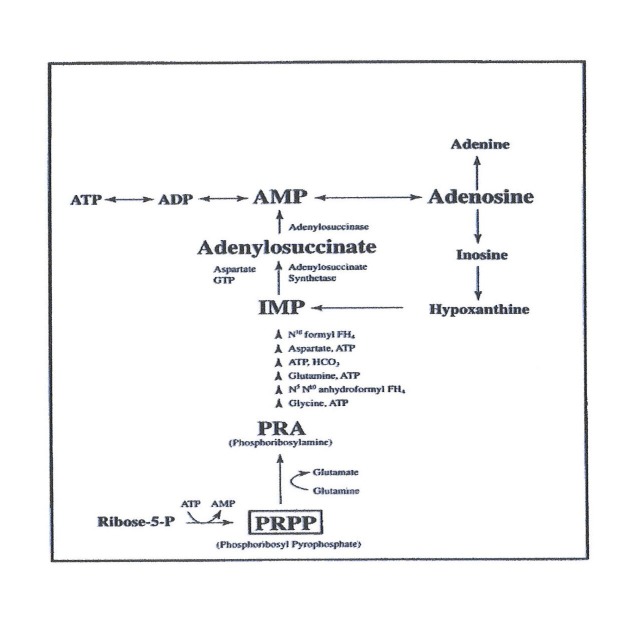
De novo pathway ADP: adenosine diphosphate; AMP: adenosine monophosphate; ATP: adenosine triphosphate; GTP: guanosine triphosphate; HCO3: bicarbonate; IMP: inosine 5'-monophosphate; PRA: phosphoribosylamine; PRPP: phosphoribosyl-pyrophosphate

Ribose enters the pentose phosphate pathway by being phosphorylated to ribose-5-phosphate by ribokinase. Ribose-5-phosphate can be utilized in different ways: for the synthesis of glucose, for glycolysis, and in the synthesis of pyrimidine nucleotides or purine nucleotides. Ribose is the substrate for the formation of PRPP, a precursor for *de novo *synthesis of ATP (Figure 3).

**Figure 4:**
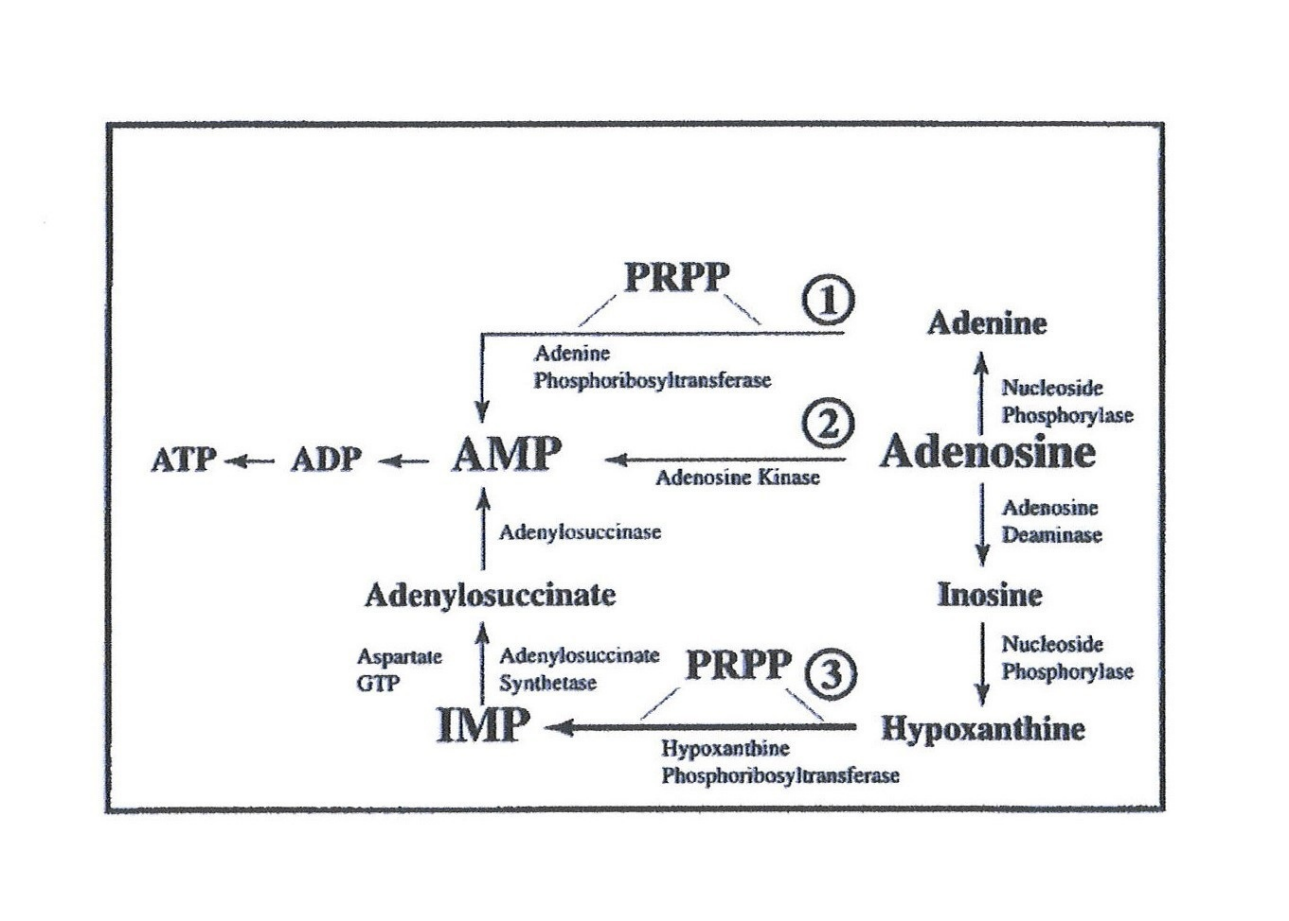
Salvage pathway ADP: adenosine diphosphate; AMP: adenosine monophosphate; ATP: adenosine triphosphate; GTP: guanosine triphosphate; IMP: inosine 5'-monophosphate; PRPP: phosphoribosyl-pyrophosphate;

In the *de novo* pathway, nucleotide bases are assembled from simpler compounds and then attached to a ribose molecule, whereas in the salvage pathway, nucleotides are synthesized by recycling intermediates in the degradative pathway for nucleotides, as well as bases, with the addition of a ribose unit (Figure 4).

There exists an interrelationship between the *de novo* and salvage pathways with PRPP playing a significant role. Ribose plays a vital role in myocardial metabolism, largely through its participation in the formation of PRPP leading to the synthesis of adenine nucleotides, mainly ATP. Supplemental ribose is unique in that it bypasses the rate-limiting enzymatic step in the pentose phosphate pathway, glucose-6-phosphate dehydrogenase, thereby directly increasing PRPP levels, leading to an enhanced production of myocardial adenine nucleotide biosynthesis. This process generates ATP molecules at an enhanced rate, which offers benefits in not only the cell’s bioenergetics status but also in the functional aspect of the cell.

D-ribose investigations

Zimmer and Gerlach found in isolated adult rat heart preparations that D-ribose supplementation produced an increased rate of adenine nucleotide synthesis following ischemia [15]. Pasque et al. also reported similar results in isolated, perfused working rat hearts subjected to 15 minutes of ischemia. D-ribose supplementation improved the recovery in myocardial ATP levels, as well as an improvement in functional recovery following ischemia [16]. Using a chronic, canine model, St.Cyr et al. reported the benefits of D-ribose supplementation when used with adenine supplementation in producing an 85% return in ATP levels by 24 hours as compared to no ATP recovery in controls following 20 minutes of global myocardial ischemia [17]. In a separate study using a chronic in vivo canine model, Schneider et al. found similar ATP benefits with D-ribose supplementation and adenine supplementation following 20 minutes of global myocardial ischemia, as well as improvements in left ventricular diastolic dysfunction (Schneider JR, St.Cyr JA, Mahoney JR, et al.: Recovery of ATP and return of function after global ischemia. Circ (Part II). 1985, 71:III 298 abstr.). In further studies using this chronic, canine model, Tveter et al. (Tveter K, St.Cyr JA, Schneider J, et al. Enhanced recovery of diastolic function after global myocardial ischemia in the intact animal. Pediatr Res. 1988, 23:226 abstr.) reported that D-ribose supplementation alone produced similar energetic and functional benefits after 20 minutes of global myocardial ischemia, as previously reported above by St.Cyr et al. and Schneider et al.

Significant coronary arterial ischemia has the potential to cause an acute myocardial infarction, which can result in functional compromise and over time can progress to the development of heart failure. The remote myocardium, adjacent but not involved in the infarcted tissue, is subjected to an increased workload, further taxing its energy supply. Zimmer et al. found a progressive decline in left ventricular systolic pressure, a decline in left ventricular dP/dTmax, an elevation in left ventricular end diastolic pressure, and lower cardiac outputs and stroke volume indices post-infarction. D-ribose supplementation post-infarction resulted in an improvement in the above measured hemodynamic parameters with stimulation in adenine nucleotide synthesis [18]. Similar findings were observed when Befera et al. supplemented D-ribose following acute myocardial infarction in adult rats. They observed an improvement in left ventricular contractility and myocardial wall thickness with less ventricular dilatation in the remote left ventricular myocardial region when supplying D-ribose (Befera N, Rivard A, Gatlin D, et al.: Ribose treatment helps preserve function of the remote myocardium after myocardial infarction. J Surg Res. 2007, 137:156 abstr.). Furthermore, when D-ribose supplementation was provided pre-infarction, Gonzales et al. reported in adult rats that there was a significant reduction in the area of the left ventricular infarct, as well as a significant improvement in left ventricular function when assessed at six hours post-infarct. Left ventricular systolic pressure and contractility were restored to normal levels with a significant improvement in left ventricular relaxation when supplementing with D-ribose [19].

The positive findings of D-ribose supplementation in ischemic cardiovascular disease in the initial pre-clinical animal studies generated interest in investigating its potential role in patients afflicted with ischemic cardiovascular diseases, both as a potential diagnostic and also as a therapeutic agent. Its diagnostic potential in identifying hibernating myocardium, found in ischemic coronary artery disease, as well as its potential benefits in patients with ischemic congestive heart failure, and during and following cardiovascular surgery have also been observed. As of today, oral D-ribose is referred to as a supplement, not requiring a prescription. Clinical patient study applications have commonly used approximately 5 gm/dose of D-ribose supplementation, multiple times/day.

Hibernating myocardium represents regional areas of myocardial dysfunction due to the prolonged hypoperfusion or ischemia. This state has the potential to be reversed upon restoration of oxygenated blood flow to these regions. Theoretically, Thallium-201 scans, dobutamine stress echocardiography, positron emission tomography, and magnetic resonance imagery can aid in the identification of these areas in the myocardium, which can provide helpful information in management strategies for revascularization. Hibernating myocardium is likely to be associated with lower levels of ATP; therefore, supplementation with an adenine nucleotide agent, such as D-ribose, might aid in increasing identification of these viable regions. Pre-clinical animal studies employing D-ribose supplementation demonstrated this benefit in enhancing the identification of these areas of hibernation. Subsequent clinical studies by Perlmutter et al. reported that D-ribose identified more reversible defects using Thallium-201 scan technology [20]. Furthermore, Hegewald et al. found that D-ribose supplementation also increased the detection of viable ischemic myocardial regions using SPECT thallium imaging [21]. More recently, Sawada et al. reported that D-ribose supplementation provided anti-ischemic effects with improving the identification of wall motion dysfunctional abnormalities found during dobutamine stress echocardiography [22].

Previously reported positive pre-clinical studies demonstrated the enhancement of D-ribose supplementation in regenerating depressed ATP levels and improved function following myocardial ischemia [1]. Pliml et al. argued that since there is significant impairment in ATP levels in the ischemic myocardium, D-ribose supplementation might offer a potential role in enhancing deficient ATP levels in patients with ischemic coronary artery disease. They investigated D-ribose supplementation in patients with stable coronary arterial disease by using serial treadmill exercise testing. D-ribose supplementation demonstrated significant benefits in increasing treadmill exercise time before the onset of angina and/or the development of ischemic electrocardiographic changes during exercise [23].

Long-term myocardial ischemia does play a factor in the development of congestive heart failure and further reports have proposed that the failing heart is energy starved [5]. Since pre-clinical animal studies have demonstrated that D-ribose supplementation enhanced the recovery of myocardial ATP levels and improved diastolic dysfunction following ischemia, Omran et al. investigated the role of D-ribose supplementation in Class II-III ischemic, congestive heart failure patients. They found an improvement in diastolic dysfunction using echocardiographic parameters. They observed an improvement in atrial contribution to left ventricular filling, a smaller left atrial chamber size, and a shortened E wave deceleration. Subjectively, these patients also experienced an improved quality of life and physical function [24]. Bayram et al. more recently demonstrated an improvement in tissue Doppler velocity, which was maintained at nine weeks in 64% of Class II-IV congestive heart failure (CHF) study patients when taking D-ribose supplementation. Approximately 45% also showed an improvement in their early diastolic filling velocity (E) to early annulus relaxation velocity (E’) [25]. Commonly, congestive heart failure patients complain of shortness of breath and fatigue, and as their failure progresses, patients develop a decrease in their ventilator efficiency. Carter et al. found that supplementation of D-ribose enabled Class II-III CHF patients with left ventricular dysfunction to maintain their maximal volume of oxygen (VO_2_max), improve their ventilatory efficiency, and experience a positive trend in their quality of life measurements (Carter O, MacCarter D, Mannebach S, et al.: D-ribose improves peak exercise capacity and ventilation efficiency in heart failure patients. J Am Coll Cardiol. 2005, 45:185 abstr). Vijay et al. concurred with these reported D-ribose benefits in a separate clinical study involving Class II-IV CHF patients when taking D-ribose supplementation. Clinically, significant improvements were observed in ventilator efficiency in the Class III-IV patients. The Class II patients were observed to have a positive, but not statistically significant, trend in improved ventilator efficiency (Vijay N, MacCarter D, Washam M, et al.: D-ribose improves ventilatory efficiency in congestive heart failure NYHA class II-IV patients. J Card Fail. 2005, 95 abstr.).

The use of D-ribose supplementation in cardiovascular surgery has revealed alterations in myocardial function in the post-revascularization period. Wyatt et al. found that the use of a cardioplegic solution of adenosine, hypoxanthine, and D-ribose supplementation intraoperatively maintained myocardial energy levels during ischemia and, with reperfusion, resulted in enhanced functional recovery post-cardiopulmonary bypass [26]. Vance et al. reported that parenteral use of D-ribose supplementation in patients undergoing elective aortic valve replacement with or without accompanying coronary artery bypass grafting resulted in the maintenance in ejection fraction one-week post-surgery, unlike the observed decline in ejection fraction in patients not receiving D-ribose supplementation (Vance RA, Einzig S, Kreisler K, et al.: D-ribose maintained ejection fraction following aortic valve surgery. FASEB J. 2000, 14:419 abstr.). More recently, oral D-ribose supplementation was added to a metabolic designed protocol for patients undergoing “off pump” coronary artery bypass revascularization. Perkowski et al. observed that the addition of D-ribose resulted in lower mortality and morbidities, along with a significant early postoperative improvement in cardiac index following revascularization [27].

## Conclusions

Cardiovascular disease still ranks as the leading cause of deaths worldwide, even with continued advances in cardiovascular therapeutic technologies. Myocardial ischemia, commonly found in cardiovascular disease, plays a major role a majority of these deaths. There continues to be strong efforts to educate the population in addressing atherosclerotic risk factors, such as refinements in a more healthy diet, regular exercise, smoking cessation, blood pressure control, and stress reduction.

All cells require adequate levels of high-energy phosphates to maintain their integrity and function. Numerous studies have demonstrated that myocardial ischemia lowers ATP levels, which can be reflected in alterations in diastolic function. Myocardial ischemia, which lowers ATP levels and causes a dysfunctional state, is a main category in cardiovascular disease. Myocardial adenine nucleotide precursor availability is an important limiting factor in the recovery of myocardial ATP molecules following ischemia, for which various substrates have been investigated. D-ribose, a naturally occurring pentose carbohydrate, has been shown in both pre-clinical and in pilot clinical studies to enhance the recovery of ATP levels and also to aid in improving left ventricular diastolic dysfunction following ischemia. These studies have shown its potential clinical benefits in acute and chronic myocardial ischemic conditions, enhancing the identification of hibernating myocardium, aiding congestive heart failure patients, and its perioperative use in cardiovascular surgery. Future, larger cohort, multicenter clinical investigations are mandatory to further substantiate these initially observed benefits in patients afflicted with ischemic cardiovascular disease.
